# Potentially inappropriate medications related to two-year progression of mild cognitive impairment and dementia

**DOI:** 10.1007/s00228-025-03977-6

**Published:** 2026-01-19

**Authors:** Hege Kersten, Maria L. Barca, Rannveig Sakshaug Eldholm, Karin Persson, Lara Thomasgaard, Keson Jaioun, Ingvild Saltvedt, Geir Selbaek, Knut Engedal

**Affiliations:** 1 The Norwegian National Centre for Ageing and Health, Vestfold Hospital Trust, Tønsberg, Norway; 2 Department of Research, Telemark Hospital Trust, Skien, Norway; 3 Institute of Clinical Medicine, Faculty of Medicine, University of Oslo, Oslo, Norway; 4 Department of Neuromedicine and Movement Science, Faculty of Medicine and Health Sciences, Norwegian University of Science and Technology, Trondheim, Norway; 5 Department of Geriatrics, St. Olavs Hospital, University Hospital of Trondheim, Trondheim, Norway; 6 Department of Geriatric Medicine, Oslo University Hospital, Oslo, Norway; 7 Division for Mental Health and Addiction, Vestfold Hospital Trust, Tønsberg, Norway

**Keywords:** Potentially inappropriate medications, Mild cognitive impairment, Dementia, Memory clinics, Dementia progression

## Abstract

**Purpose:**

To document use and impact of potentially inappropriate medications on two-year progression of dementia in individuals with cognitive declines.

**Methods:**

A retrospective study of 397 patients with Mild Cognitive Impairment (MCI) or dementia diagnosed and followed-up in outpatient memory clinics in Norway during 2009 − 18. Beers (2019)- and STOPP-2 criteria were used to identify Potentially Inappropriate Medications (PIMcogs) in individuals with cognitive impairments at baseline and two-year-follow-up. PIMcog use in terms of dementia severity, cognitive function, and neuropsychiatric and depressive symptoms were analyzed in regression models.

**Results:**

The prevalence of PIMcogs increased from 16% at baseline to 23% at follow-up. PIMcog users were more likely to be women (63.5%), and they used more drugs, with a median of 5 drugs at baseline and 4 drugs at follow-up, compared to non-users who had a median of 3 used drugs at both time points. PIMcog users had higher median Neuropsychiatric Inventory severity sum scores (6 [3.0–11.0] versus 4.0 [2.0–7.0]) and median Cornell Scale for Depression in Dementia scores (6.5 [3.0–11.0] versus 4.0 [1.0–7.0]) compared to non-users at follow-up (*p* ≤ 0.002). PIMcog users exhibited more severe dementia, with a Clinical Dementia Rate-Sum of Boxes (CDR-SB) score of 7.0 (4.0–13.0) compared to 6.0 (3.5–10.0) in non-users. The median annual increase in CDR-SB was one unit, and PIMcog use at follow-up was significantly associated with more rapid progression of dementia severity.

**Conclusion:**

Faster dementia progression was documented among PIMcog users although, the prevalence of PIMcogs was generally low in Norwegian memory clinic patients with cognitive impairments.

**Supplementary Information:**

The online version contains supplementary material available at 10.1007/s00228-025-03977-6.

## Introduction

Several drugs are available to slow the progression of dementia [1]. Conversely, could the use of CNS-depressing drugs accelerate dementia progression? The prevalence of dementia is rising rapidly, and conducting research to prevent the transition from mild cognitive impairment (MCI) to dementia, as well as slowing the progression of dementia, are key priorities for global public health [2].

Avoiding medications that may exacerbate cognitive impairment and accelerate dementia progression is a crucial aspect of dementia and MCI management. Many consensus-based tools have been developed to avoid prescribing of potentially inappropriate drugs (PIMs) to older adults, with the Screening Tool of Older Person’s Prescriptions (STOPP) and the American Geriatric Society Beers Criteria^®^ being among the most widely used [3, 4]. According to STOPP and Beers criteria, CNS-depressants such as benzodiazepines (BZDs), BZD-related drugs, certain antipsychotics, and drugs with strong anticholinergic effects are considered potentially inappropriate medications for people with cognitive disorders (PIMcogs) [5–7]. This inappropriateness is due to drug–disease interaction, which can exacerbate cognitive impairment and cause a wide range of serious adverse effects, including increased risks of all-cause mortality, stroke, vascular death, myocardial infarction, falls, and further cognitive decline [8–11]. Despite their risks, PIMcogs are still commonly prescribed for managing neuropsychiatric symptoms in dementia. In the USA, 82% of the patients referred to a hospital for concerns of cognitive decline, used sedatives and anticholinergic drugs [11]. Other studies have shown a 20–30% prevalence of PIMcogs in community-dwelling older adults with cognitive disorders [8, 12]. A systematic review, -including 11 studies from memory clinics in eight different countries, -found PIM -and polypharmacy rates of 38% and 60%, respectively, with significant variation between countries [13]. Prescription patterns, drug use, and PIMcog prevalence depend on local pharmacotherapeutic practices and there is a need for studies from memory clinics in northern Europe, where the prescription rate is generally lower.

The relationship between PIMcog use and dementia risk is documented in many studies and long-term exposure to PIMcogs has been associated with elevated risk of dementia and MCI [14, 15]. However, the findings are inconsistent, and the evidence of this association is weak [16]. The impact of PIMcogs on the course and progression of cognitive decline and dementia is much less documented. But, a French study showed a steeper cognitive decline over almost 3 years, -measured by Mini Mental State Examination, -in memory clinic patients with moderate and high anticholinergic and sedative drug burden [17]. The influence of PIMcog use on the progression of other disease related factors such as neuropsychiatric symptoms and dementia severity is not yet documented. Therefore, further research, particularly longitudinal studies, is needed to assess the impact of PIMcog use on the progression in several disease related disease factors in dementia and MCI.

The aim of the present study was to document the use of PIMcogs and their impact on disease progression assessed by changes in dementia severity, neuropsychiatric – and depressive symptoms, and cognitive function in patients with MCI and dementia, diagnosed and followed up in Norwegian memory clinics over two years.

## Methods

### Study design

This study presents a retrospective analysis of pooled data from patients recruited between 2009 and 2016 in four longitudinal observational studies conducted in Norway. Only participants with two-year follow-up assessments and complete drug records at both baseline and follow-up were included in the current analysis. Three studies were part of the ‘Progression of Alzheimer’s Disease and Resource Use (PADR)’ project, which investigated various aspects of disease progression in AD patients, from diagnostic workup (baseline) to follow-up after a mean of 24 months. The fourth study was a two-year prospective Nordic cohort study on quality of life and specific needs in individuals with Young Onset Dementia (YOD) and their families (ClinicalTrials.gov identifier: NCT02055092) [18]. Only the patients from the Norwegian part of the YOD-study were enrolled in the present analysis.

Participants were recruited from eight centers participating in the Norwegian Registry of Persons Assessed for Cognitive symptoms (NorCog). NorCog is a national registry that compiles data from standardized cognitive assessments across Norway [19]. The participating centers are interdisciplinary outpatient memory clinics specializing in dementia diagnosis.

### Study population and diagnostic workup

We merged data from 335 participants in the PADR project with data from 62 patients in the YOD study, resulting in a total of 397 participants in the current database. All participants were living at home, had a proxy available to provide information, and had been referred to memory clinics for assessment. In accordance with the inclusion criteria for the YOD study, participants were diagnosed with either frontotemporal dementia (FTD) or Alzheimer’s dementia (AD) prior to inclusion, while participants in the PADR project were diagnosed with MCI or any form of dementia at baseline. During the diagnostic workup, the patients underwent physical examinations and neuropsychological assessments with standardized scales. Structural brain imaging (magnetic resonance imaging (MRI in most cases, otherwise CT scan) was performed and analyzed. Blood samples and cerebrospinal fluid were collected from some of the PADR-patients according to clinical indication before diagnosis by the consultants at each memory clinic. Dementia diagnoses were made according to the ICD-10 criteria [20], and the Winblad criteria for MCI diagnosis [21]. The National Institute of Neurological and Communicative Disorders and Stroke-Alzheimer’s Disease and Related Disorders Association (NINCDS-ADRDA) criteria were used for Alzheimer’s dementia (AD) [22]. Positive biomarkers for Aβ and/or neuronal injury (medial temporal atrophy on MRI and/or elevated levels of CSF p-tau) supported the diagnosis of AD. The Neary and/or the International consensus criteria were used for behavioral variant FTD, and the Mesulam criteria were used for the language variant [23–25] Vascular dementia, mixed dementia, lewy body dementia, Parkinson’s dementia and unspecified dementia are categorized as other dementia. The diagnoses were reconsidered by at least one of the research physicians reviewing all available data from the baseline examination.

### Data collection

Data were retrieved from case report forms (CRFs) recorded at baseline and follow-up. The baseline records included sociodemographic characteristics and diagnostic workup results, while structured clinical interviews with patients and caregivers, as well as cognitive and neuropsychiatric assessments, were recorded at both time points.

The sociodemographic variables included in the analysis were age at baseline, age at symptom onset (≤ 65 years or > 65 years), sex, level of education, and living situation. All participants lived at home at baseline, and we separated the group by whether they lived alone or together with relatives. At follow-up, these two groups were merged and compared with those in institutional residency. Age at symptom onset was reported by patients and their relatives during baseline structured interviews.

Data on used drugs were prospectively recorded from referral letters and information provided by patients and caregivers at both time points. Unfortunately, data on drug dosages and pro re nata (PRN) medications were insufficiently recorded. Thus, all analyses are based on regularly prescribed medications. Drugs were classified according to the Anatomical Therapeutic Chemical (ATC) classification system prior to analysis.

### Polypharmacy and potentially inappropriate medications in individuals with cognitive impairment (PIMcogs)

Polypharmacy was defined as concomitant use of ≥ 5 drugs, while hyperpolypharmacy was defined as concomitant use of ≥ 10 drugs. This is the most commonly used numeric definitions of polypharmacy and hyperpolypharmacy [26]. Drugs that, according to the Beers 2019 and the STOPP-2 criteria should be avoided in people with cognitive impairment were defined as PIMcogs [5, 6]. Consequently, Benzodiazepines (BZD), BZD-derivatives, and BZD-like hypnotics, antipsychotics and other drugs with strong anticholinergic properties were categorized as PIMcogs [27]. Antipsychotics listed as anticholinergic drugs in Beers 2019, were registered as anticholinergics. For a complete list of PIMcogs, see Supplementary Appendix 1. Updated versions of Beers and STOPP criteria were published in 2023, but since drug use in this study was recorded through 2018, we used the Norwegian STOPP-2 version and the 2019 Beers Criteria and adapted it to the Norwegian drug market.

### Stage of dementia, cognitive function and neuropsychiatric symptoms

Dementia severity was evaluated by summing the six domains of the clinical dementia rate scale (CDR): memory, orientation, judgment and problem solving, community affairs, home and hobbies, and personal care. This summation is referred to as the CDR-sum of boxes (CDR-SB) and ranges from 0 to 18, with higher scores denoting more severe impairment [28]. Cognitive functioning was measured by the Norwegian revised version of Mini Mental State Examination (MMSE-NR) ranging from 0 to 30, with lower scores denoting more severe impairment [29]. Neuropsychiatric symptoms were measured by the severity sum score of the Neuropsychiatric Inventory (NPI), a questionnaire representing the severity of the individual neuropsychiatric symptoms. The NPI sum score ranges from 0 to 36, with higher scores denoting more severe neuropsychiatric symptom [30]. Depressive signs and symptoms were measured by the Cornell Scale for Depression in Dementia (CSDD), based on interviews with the patients and their informants. CSDD has 19 items over 5 categories and each item is assessed for severity between 0 and 2 [31]. Scores totalling 7 point or more is indicating possible depression [32].

### Statistical analyses

The study population was stratified by use and non-use of PIMcogs. Additionally, those using PIMcogs at both baseline and follow-up were separated into one group for possible sub-analyses. The distribution of continuous variables across the two study groups was examined using distribution plots. The normality of continuous variables was assessed using the Shapiro‒Wilk test. As all variables were non-normally distributed, the data are presented as medians with interquartile ranges (IQRs). Study groups were compared using Mann‒Whitney U tests for the continuous variables. For categorical variables, Pearson’s chi-square (χ²) test), Yates continuity, and Fisher’s exact test were used as appropriate.

Drug use was registered at baseline and follow-up, and Related Samples Wilcoxon Signed-Rank test was used to compare the drug use at both time points. Spearman correlations between patients’ characteristics and PIMcog use were inspected before univariate logistic regression analyses were performed with PIMcog use at each visit as dependent variables. We chose logistic regression as we were interested in patient characteristics associated with the use of any PIMcogs at both baseline and follow-up. Independent variables associated with PIMcog use with significance level *p* ≤ 0.1 in the single variable analyses, were added into the multivariable models. Correlations between the independent variables were checked with Spearman correlations with a level of acceptance for correlation r ≤ [0.5]. The linearity related to the log odds were checked for the continuous independent variables before adding into the multivariable models.

Progression in disease-related factors were calculated by change from baseline to follow-up (*△ NPI*=$NPI_{follow-up}$-$NPI_{baseline}$; *△ Cornell*=$Cornell_{follow-up}$-$Cornell_{baseline}$; *△ MMSE*-*NR*=*MMSE*-$NR_{follow-up}$-*MMSE*-$NR_{baseline}$; $\triangle CDR\_SOB$=$CDR\_SOB_{follow-up}$-$CDR\_SOB_{baseline}$) and divided by time (years) between the two visits. The associations between the number of PIMcogs used and the progression in disease-related factors were analyzed with univariate and multivariate linear regression models. Pearson correlations between covariates and dependent variables were assessed before covariates were added into the multivariate model. The linear models were assessed for independence of errors and multicollinearity with an acceptable correlation of variance inflation factor (VIF) ≤ 1.5. The beta coefficients were reported with 95% CIs.

To reduce the risk of type 1 error, we performed additional subanalyses. We categorized disease progression by threshold values for increment in CDR-SB per year: Slow progressors < 1 unit/year, intermediate progressors 1- < 2 units/year and rapid progressors ≥ 2 units/year [33]. We investigated the association between PIMcog use and intermediate/rapid disease progressors in binary logistic regression models.

Statistical significance was set at 5%. IBM SPSS Statistics version 29.0 (IBM Corp, Armonk, NY, USA) and STATA version 18.0 (STATAcorp, College Station, TX, USA) were used for all statistical analyses.

### Ethics

Data for this study were retrieved from baseline and follow-up assessments conducted in the PADR-project approved by the Regional Committee for Medical and Health Research Ethics for South-East Norway A (REC number 2011/531), and the YOD-project approved by the Regional Committee for Medical and Health Research Ethics for Northern Norway (REC number 013/2141). All patients received oral and written information about the studies and voluntary gave written, informed consent to participate prior to baseline assessments. Only patients with capacity to consent were recruited at baseline. No additional tests were conducted in the present study and according to the Regional Committees for Medical and Health Research Ethics for South-East Norway D (REC number 2015/410), no further Consent to Participate Declaration was required for the participants in the present study The data collection and exchange of information from the PADR -and YOD-project, along with the current study protocol, was approved by Regional Committees for Medical and Health Research Ethics for South-East Norway D (REC number 2015/410).

## Results

### Characteristics

At baseline, the study cohort (*n* = 397) had a median age of 71 years, with an equal distribution of men and women, and a median of 12 years of education. Nearly one third (33%) of the participants were diagnosed with mild cognitive impairment (MCI) and 51% were diagnosed with Alzheimer’s dementia. After two years, 64 patients with MCI at baseline had converted to dementia and 16% had MCI at follow-up.

At baseline, 16% of participants (*n* = 63) were using PIMcogs, with a significantly higher prevalence among females (20%) compared to men (11%). At the two-year follow-up, the proportion of PIMcog users increased to 23% (*n* = 90), with no significant gender differences. The prevalence of polypharmacy and the median number of total drugs used was significantly higher among PIMcog users compared to non-users at both time point (*p* < 0.001), and at follow-up 70% of the PIMcog users used ≥ 5 drugs concomitantly.

The prevalence of PIMcogs among individuals with MCI or dementia was similar at both time points (*p* ≥ 0.812). We included 62 participants from a study of individuals with young-onset FTD or AD. Consequently, 40% of participants in our study cohort reported their age of symptom debut to be ≤ 65 years, and 6.3% were diagnosed with FTD. There were no differences in PIMcog prevalence between the different dementia diagnoses (Table 1).

By the two-year follow-up, more than one-fifth of participants were residing in institutions, and their use of PIMcogs was not significantly higher than that of those living at home (*p* > 0.215).

**Table 1:** Characteristics of the study cohort stratified by use and non-use of PIMcogs at baseline and follow-up. Continuous variables presented with median and (Interquartile Range) and categorical variables with frequency and (Percentage)

Characteristics		Baseline			Two years follow-up		
		PIMcog users *n*_1_ = 63	PIMcog non-users *n*_2_ = 334	*p* ^*^	PIMcog users *n*_1_ = 90	PIMcog non-users *n*_2_ = 307	*p* ^*^
Age		71.0 (63.0–78.0)	72.0 (64.0–79.0)	0.548	73.0 (65.0–80.0)	74.0 (66.8–80.0)	0.559
Age of symptom debut	**≤** 65	19 (30.2)	138 (41.3)	0.097	32 (35.5)	125 (40.7)	0.379
	> 65	44 (69.8)	196 (58.7)		58 (64.4)	182 (59.3)	
Sex	Female	40 (63.5)	160 (47.9)	**0.023**	53 (58.9)	147 (47.9)	0.066
Years of education		*n*_*1*_ = 62 12.0 (8.0–5.0)	*n*_*2*_ = 327 12.0 (9.0–15.0)	0.527	*n*_*1*_ = 89 12.0 (9.0–15.0)	*n*_*2*_ = 300 11.0 (8.0–15.0)	**0.049**
Living situation	Alone	18 (28.6)	98 (29.3)	0.506	66 (73.3)^**^	244 (79.5)^**^	0.215
	With relatives	45 (71.4)	236 (70.7)				
	Institution	0	0		24 (26.6)	63 (20.5)	
Number of drugs used		5.0 (3.0–6.0)	3.0 (1.0–4.0)	**< 0.001**	4.0 (2.0–6.0)	3.0 (1.0–4.0)	**< 0.001**
Poly- pharmacy	≥ 5 drugs	28 (44.4)	70 (21.0)	**< 0.001**	63 (70.0)	103 (33.5)	**< 0.001**
	≥ 10 drugs	6 (9.5)	5 (1.5)	**< 0.001**	10 (17.5)	12 (3.9)	**< 0.001**
Mild Cognitive Impairment		20 (31.7)	109 (31.1)	0.890	14 (15.6)	51 (16.6)	0.812
Dementia		43 (68.3)	225 (67.4)		76 (84.4)	256 (83.4)	
Alzheimer’s dementia		28 (44.4)	173 (51.8)	0.284	59 (65.6)	195 (63.5)	0.723
FTD		5 (5.6)	20 (6.0)	0.559	4 (4.4)	21 (6.8)	0.411
Other dementia (VasD, MixD, LBD, PD, Unspec)		26 (8.5)	16 (17.8)	0.012	13 (14.4)	40 (13.0)	0.729

*FTD* Frontotemporal Dementia, *LBD* Lewy Body Dementia, *PD* Parkinson’s Dementia, *VasD* Vascular Dementia, *MixD* Mixed Dementia, Unspec = Unspecified dementia

^*^P-value based on Mann Whitney U test, Pearson Chi-square test, Yates continuity, and Fisher exact test as appropriate. *P*-values < 0.05 in bold font. ^**^Living at home

### Drug use and PIMcog prevalence

The participants used from 0 to 18 drugs concomitantly, with acetylsalicylic acid (ASA) being the most commonly used. The prevalence of polypharmacy and PIMcogs increased significantly from baseline to 42% and 23%, respectively, at follow-up (*p* < 0.001) There were 63 participants using 1–3 PIMcogs at baseline and 90 participants using 1–4 PIMcogs at follow-up (Table 2). There were 19 PIMcogs with different ATC codes in use, used 76 times, at baseline and 25 PIMcogs with different ATC code in use, used 127 times, at follow up (Supplementary Appendix 1).

Among the 90 PIMcog users at follow-up, 35 also used PIMcogs at baseline, 28 persons had discontinued PIMcogs before follow-up, and 55 had started PIMcogs between baseline and follow-up.

The prevalence of hypnotics/benzodiazepines increased significantly (*p* < 0.001) (Table 2), and 10 participants used more than one hypnotic/benzodiazepine in combination at follow-up. Zopiclone was the most frequently used PIMcog at both time points, with 21 users at baseline and 35 users at follow-up. The use of antipsychotics also increased from baseline to follow-up with risperidone and quetiapine being the most frequently started medication. The number of anticholinergic drugs were quite stable and the most used was solifenacin, prescribed to nine participants at both baseline and follow-up.

A significantly higher proportion of participants (39%) were using antidementia drugs at follow-up. Among users of antidementia drugs (N06D), 147 participants were prescribed monotherapy with acetylcholinesterase inhibitors, while 8 patients were using a combination of antidementia drugs. Of particular concern, two patients used the inappropriate combination of an acetylcholinesterase inhibitor and an anticholinergic drug at baseline, and this had increased to eight patients at follow-up.

**Table 2:** Drug use and PIMcog prevalence at baseline and follow-up

Drug class		Baseline (*n*=397)	Two years follow-up (*n*=397)	Difference from baseline to follow-up (*p*-value)*
Total number of drugs, median (IQR)		3.0 (1.0–5.0)	4.0 (2.0–6.0)	**< 0.001**
Polypharmacy (≥ 5 drugs), *n* (%)		98 (24.7)	166 (41.8)	**< 0.001**
Antipsychotics, *n* (%)		8.0 (2.0)	21.0 (5.3)	**0.003**
Hypnotics/benzodiazepines, *n* (%)		26.0 (6.5)	56.0 (14.1)	**< 0.001**
Anticholinergics, *n* (%)		34.0 (8.6)	31.0 (7.8)	0.622
Number of PIMcogs, *n* (%)	0	334.0 (84.1)	307.0 (77.3)	
	1	52.0 (13.1)	62.0 (15.6)	
	2	10.0 (2.5)	20.0 (5.0)	
	3	1.0 (0.3)	7.0 (1.8)	
	4	-	1.0 (0.3)	
Use of ≥ 1 PIMcog, *n* (%)		63.0 (15.9)	90.0 (22.7)	**< 0.001**
Antidementia drugs, *n* (%)		63.0 (15.9)	155.0 (39.0)	**< 0.001**

^*^P**-**value based on Related Samples Wilcoxon Signed-Ranks Test. Values < 0.05 in bold font

### Factors associated with PIMcog use

The univariate logistic regression analyses showed that there is a strong association between the number of drugs used and the risk of using a PIMcog. At baseline, the odds of using a PIMcog increased with 1.31 for each drug added to the medication list (Table 3). Having polypharmacy and being a female increased the risk of PIMcog use at both baseline and follow-up (*p* ≤ 0.039), while age, education, dementia diagnosis and living in an institutionalization did not. As polypharmacy and the number of total drugs used were strongly correlated, the polypharmacy variable was excluded from the multivariable models. In the multivariable models, females and the number of total drugs used remained significantly associated with PIMcog use at both time points (*p* ≤ 0.039). At two-years follow-up, the chance of using a PIMcog was 1.7 times higher for females than for men and for every drug added to the medication list, the odds of using a PIMcog increased with 1.36 (Table 3).

**Table 3:** Logistic regression models for factors associated with PIMcog use at baseline and follow-up

Independent variables	PIMcog use baseline *n* = 63						PIMcog use follow-up *n* = 90					
	Univariate			Multivariate			Univariate			Multivariate		
	OR	95% CI	*p*	OR	95% CI	*p*	OR	95% CI	*p*	OR	95% CI	*p*
Age (years)	1.01	(0.98–1.04)	0.415				1.01	(0.99–1.04)	0.374			
YOD	0.61	(0.34–1.10)	0.099	1.50	(0.81–2.77)	0.200	1.25	(0.76–2.03)	0.379			
Female (ref: men)	1.89	(1.09–3.30)	**0.025**	1.87	(1.043–3.34)	**0.035**	1.56	(0.97–2.51)	**0.067**	1.74	(1.03–2.94)	**0.039**
Years of education	1.03	(0.96–1.11)	0.391				0.96	(0.90–1.03)	0.274			
Living alone (ref: with comparent)	0.96	(0.53–1.75)	0.90									
Living in institution (ref: home)							1.41	(0.82–2.42)	0.217	1.68	(0.92–3.1)	0.093
No. of drugs	1.31	(1.19–1.44)	**< 0.001**	1.30	(1.18–1.44)	**< 0.001**	1.35	(1.24–1.48)	**< 0.001**	1.36	(1.25–1.49)	**< 0.001**
Polypharm ≥ 5 drugs	3.02	(1.72–5.26)	**< 0.001**				4.62	(2.78–7.69)	**< 0.001**			
Dementia (ref: MCI)	1.04	(0.585–1.86)	0.890				1.09	(0.65–1.80)	0.750			

*OR* Odds Ratio, *CI* Confidence Interval, *YOD* Young Onset Demetia defined as age of symptom debut ≤ 65 years, *No* Number, *Polypharm* Polypharmacy, *MCI* Mild Cognitive Impairment

## Dementia-related disease factors at baseline and follow-up

There were no significant differences (*p* ≥ 0.37) in clinical dementia severity, cognitive measures, or depressive and other neuropsychiatric symptoms between the study groups at baseline. At the two-year follow-up, PIMcog users exhibited significantly more depressive and neuropsychiatric symptoms compared to non-users and had more severe dementia (*p* ≤ 0.030) (Table 4).

**Table 4:** Comparisons of dementia-related disease factors between users and non-users of PIMcogs at baseline and follow-up. Data presented as median and (Interquartile Range)

Assessments	Baseline				Two-years follow-up			
	Total ***n*** **= 397**	PIMcog users *n*_1_ = 63	PIMcog non-users *n*_2_ = 334	*p*-value*	Total ***n*** **= 397**	PIMcog users *n*_1_ = 90	PIMcog non-users *n*_2_ = 307	*p*-value*
Clinical dementia rates-sum of boxes (CDR-SB) (0–18)	*n =* 397 4.0 (2.0–5.8.0.8)	*n*_*1*_ = 63 4.0 (2.0–6.0)	*n*_*2*_ = 334 4.0 (2.0–5.5.0.5)	0.371	*n* = 393 6.0 (3.5–11.0)	*n*_*1*_ = 89 7.0 (4.0–13.0)	*n*_*2*_ = 304 6.0 (3.5–10.0)	**0.030**
Mini Mental State Examination- Norwegian revised version (MMSE-NR) (0–30)	*n* = 389 25.0 (21.0–27.0)	*n*_*1*_ = 62 25.0 (21.0–27.3.0.3)	*n*_*2*_ = 327 25.0 (21.0–27.0)	0.929	*n* = 358 22.0 (17.0–26.0)	*n*_*1*_ = 77 23.0 (15–27.5.5)	*n*_*2*_ = 281 22.0 (17.0–26.0)	0.996
Cornell score (0–36)	*n* = 370 5.0 (2.0–8.3.0.3)	*n*_*1*_ = 59 5.0 (2.0–9.0)	*n*_*2*_ = 311 5.0 (2.0–8.0)	0.394	*n* = 380 4.0 (2.0–8.0)	*n*_*1*_ = 88 6.5 (3.0–11.0)	*n*_*2*_ = 292 4.0 (1.0–7.0)	**< 0.001**
Neuropsychiatric Inventory (NPI) severity sum score (0–36)	*n =* 345 4.0 (2.0–8.0)	*n*_*1*_ = 56 4.5 (1.0–8.0)	*n*_*2*_ = 289 4.0 (2.0–8.0)	0.452	*n* = 381 4.0 (2.0–8.0)	*n*_*1*_ = 86 6.0 (3.0–11.0)	*n*_*2*_ = 295 4.0 (2.0–7.0)	**0.002**

^*^P-value based on Mann Whitney U test. Values < 0.05 in bold font

## The impact of PIMcog use on the progression of dementia-related disease factors

Table 5 shows the median change in the disease-related factors from baseline to follow-up for the whole study cohort. We found a median progression in CDR-SB of 1 point increase per year, corresponding to slow-intermediate progression in Alzheimer’s Dementia.

The impact of PIMcog use on disease progression was analyzed by linear regression models and the unadjusted beta coefficients with 95% CI are shown in Table 5. The number of PIMcogs used at follow-up was significantly associated with the progression of CDR-SB per year, *p* = 0.006, but did not associate with other disease-related factors. The dichotomized variable use/no use of PIMcogs was also significantly associated with CDR-SB per year (*p* = 0.025). No relation between the use of PIMcogs at both baseline and follow-up (*n* = 35), and disease-related factors were identified in the subanalyses performed (*p* ≥ 0.364).

**Fig. 1:**
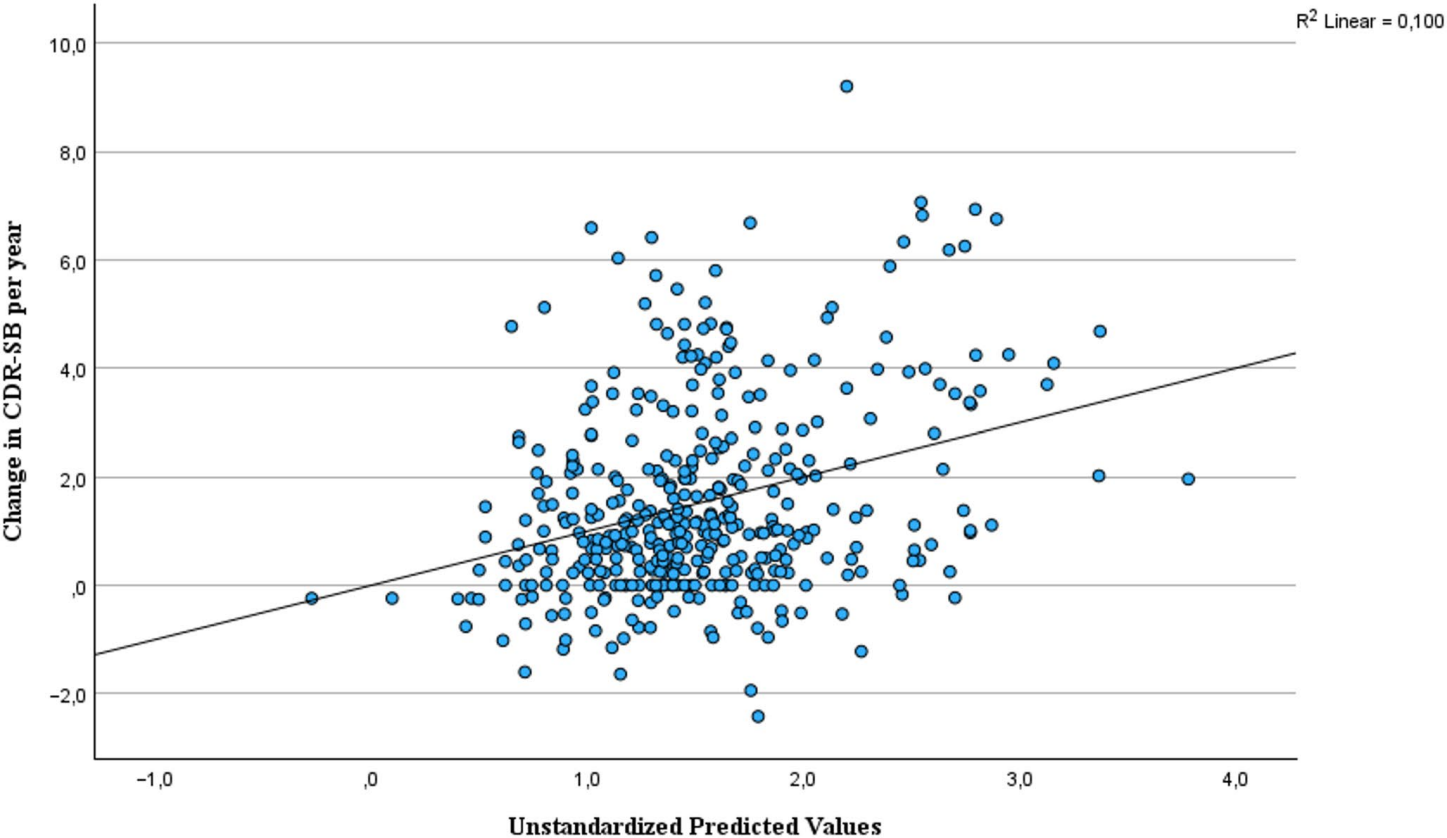
Multiple regression model showing the adjusted association between PIMcog use at follow-up and change in CDR-SB from baseline to follow-up

The association found between the number of PIMcogs used at follow up and the progression in CDR-SB were analyzed further in a multivariate linear regression analysis with ∆ CDR-SB/years between baseline and follow-up as dependent variable. The covariates added to the multivariable model were age, the total number of drugs used, and living in an institution, all correlated to the progression in CDR-SB with a Person correlation coefficient ≥ 0.117, *p* ≤ 0.010. With the variance inflation factors for the covariates ≤ 1.259, collinearity was not found to be a problem in this model. The multivariable model explained 10% of the variance in the change in CDR-SB per year (*p* < 0.001). The association between the number of PIMcogs used at follow-up and the progression in CDR-SB did not remain statistically significant after adjusting for the covariates (B = 0.262, (−0.021, 0.545), *p* = 0.069). The two factors most strongly associated with ∆ CDR-SB/years were living in an institution (B = 1.030, *p* < 0.01) and higher age (B = 031, *p* < 0.01), while the number of drugs used was no longer significant (*p* = 0.358) in the multivariable model. Figure 1 shows a scatterplot of the multivariable regression model and the prediction of change in CDR-SB per year.

Finally, a binary logistic regression model was performed to investigate the association between PIMcog use at follow-up and intermediate/rapid disease progressors (*n* = 120), defined as ≥ 1.0 unit increase in CDR-SB. We found that using PIMcogs at follow up was significantly associated with intermediate/rapid disease progression. The PIMcog users had 1.77 times higher risk of being an intermediate/rapid progressor than being a slow disease progressor (OR = 1.77, CI: 1.08, 2.89, *p* = 0.023).

**Table 5:** Multiple univariate linear regression analysis for associations between PIMcog use and the progression in disease-related factors from baseline to follow-up

Dependent variables Disease-related factors	*n*	Median ∆ (IQR)/time*	Number of PIMcogs used at baseline		Number of PIMcogs used at follow-up	
			Beta coefficient (95%CI)	*p*	*Beta coefficient (95%CI)*	*p*
Clinical Dementia Rate Sum of Boxes	393	1.0 (0.3, 2.3)	0.005 (−0.021, 0.031)	0.700	0.051 (0.015, 0.088)	**0.006**
Cornell sum score	358	0.0 (−1.0, 1.0)	0.004 (−0.014, 0.023)	0.626	0.021 (−0.006, 0.048)	0.120
Neuropsychiatric Inventory sum of severity score	300	0.0 (−1.0, 2.0)	−0.001 (−0.019, 0.016)	0.867	−0.009 (−0.035, 0.016)	0.476
Mini Mental State Examination Sum score -Norwegian revised version	376	−1.0 (−2.7, 0.0)	0.000 (−0.018, 0.18)	0.966	−0.017 (−0.042, 0.008)	0.181

*$\triangle CDR\_SOB$=$CDR\_SOB_{follow-up}$-$CDR_{SOB\;baseline}$; *△ Cornell*=$Cornell_{follow-up}$-$Cornell_{baseline}$; *△ NPI*=$NPI_{follow-up}$-$NPI_{baseline}$; *△ MMSE*=$MMSE_{follow-up}$-$MMSE_{baseline}$. All ∆s are adjusted for years between baseline and follow-up

*IQR* Inter Quartile Range, *CI* Confidence interval

## Discussion

We identified that the prevalence of PIMcogs reached 23% at two-years follow-up and higher PIMcog use was associated with a steeper progression of dementia. PIMcog users at follow-up had significantly higher severity stage of dementia and exhibited more neuropsychiatric and depressive symptoms compared to non-users.

The prevalence of PIMcogs and the overall drug use were lower than previously reported from memory clinics in other countries [17, 27]. Still, the fact that almost one fourth of our patients used medications that may interfere with cognitive function is concerning, as adverse drug effects could impact the accuracy of diagnostic assessments and the progression of cognitive decline.

We found that 25% had polypharmacy at baseline, and 42% were exposed to polypharmacy at the two-year follow-up, while a systematic review reported the pooled prevalence of polypharmacy among cognitively impaired memory clinic outpatients to be 60% [13]. The review included 11 studies from eight countries, but only four of the studies were from European countries, and none were from northern Europe. The lower medication consumption in our cohort could be explained by the participants’ younger median age of 71 years and the earlier stage of the disease compared to other memory clinic cohorts [13, 17, 27]. The generally low drug use among Norwegian memory clinic patients could partly explain the low prevalence of PIMcogs (16%−23%), but as many PIMcogs are used as needed and not registered as regularly used drugs, it is likely the actual prevalence of PIMcogs is higher than currently documented.

It is well known that the risk of using inappropriate drugs rises as the medication consumption increases. Accordingly, we found that an increasing number of drugs and the presence of polypharmacy significantly predicted the use of PIMcogs. Being a female was also significantly associated with higher PIMcog use, while other well-known risk factors such as higher age and institutionalization were not associated with inappropriate drug use. We found that the use of benzodiazepines (BZDs) increased significantly from baseline to follow-up, primarily due to the increased use of z-hypnotics. This increment is expected as the disease progresses, and has been previously reported in a study using data from the Norwegian Prescription Database [34]. The low use of anticholinergic drugs (< 9%) slightly decreased from baseline to follow-up and could partly be explained by physicians’ increased focus on deprescribing these drugs. In contrast, previous studies from other countries have shown an increased risk of receiving anticholinergic drugs as dementia progresses [35]. Almost 40% of participants were using antidementia drugs at follow-up, and eight individuals were prescribed the inappropriate combination of an acetylcholinesterase inhibitor and an anticholinergic drug. This is a lower number than reported in other pharmacoepidemiologic studies; however, the concomitant use of these drugs can lead to a pharmacodynamic drug-drug interaction, which should be completely avoided, as the anticholinergic drug may counteract the modest symptomatic benefits of acetylcholinesterase therapy [35, 36].

Comparing PIMcog users and non-users at follow-up, we found that the dementia severity was significantly worse among the PIMcog users. PIMcog users also reported significantly more neuropsychiatric and depressive symptoms than non-users. The interpretation of these cross-sectional findings two years after baseline is confounded by the indications for the PIMcog prescriptions. We likely identified a subgroup of dementia patients with more neuropsychiatric symptoms of dementia, which prompted their use for PIMcogs, rather than identifying a causal link between PIMcog use and worsening dementia. As we did find more neuropsychiatric symptoms among the PIMcog users, this could also be understood as alignment to the many reports of limited efficacy of pharmacologic treatment of neuropsychiatric symptoms of dementia [37]. Considering the reasons for PIMcog prescribing and exploring whether there are specific patient profiles that are more likely to receive PIMcogs, could be useful in future studies.

An increasing number of PIMcogs used at follow-up was significantly associated with a steeper annual dementia progression as measured by CDR-SB. We also found that using PIMcogs at follow-up significantly predicted intermediate/rapid disease progression, defined as ≥ 1.0 unit increase in CDR-SB. These findings align with a recent study showing a steeper cognitive decline among memory clinic patients with a moderate to high anticholinergic and sedative drug burden [17]. In contrast, a 2011 study found no effect of anticholinergic drugs on cognitive deterioration over 18 months in people with Alzheimer’s dementia [38]. The contrasting findings may be explained by differences in the study cohorts’ characteristics. The participants in the 2011 study were older and had more severe dementia. Additionally, anticholinergic load was measured using the Anticholinergic Burden Scale, which includes drugs with weak anticholinergic effects. Although, it is primarily cumulative exposure to strong anticholinergic drugs and the most lipophilic benzodiazepines that has been associated with irreversible cognitive decline and increased risk of dementia, and particularly in younger people with milder-stage dementia [14, 39].

Disease progression is generally slower in the initial stages of dementia [33]. We found a median annual change of one unit increment in CDR-SB and one unit reduction in MMSE over the two-year study period. PIMcog users had a higher risk of more rapid progression, compared to non-users. However, PIMcog use at follow-up did not influence change in the cognitive assessment, MMSE. This lack of association may be due to the selection of individuals with MCI and milder stages of dementia in the present cohort, as MMSE is most sensitive to changes in the middle stages of the disease [40]. In contrast, CDR-SB captures changes across all stages of the disease, and we therefore believe this assessment is the most reliable measure of disease progression, given the characteristics of our study participants [33].

The identified association between PIMcog use and rapid dementia progression could be interpreted as interactions between the central nervous system effects of PIMcogs and neurodegenerative disease processes that accelerate dementia progression. However, reverse causality is also a plausible explanation for the observed association. A recently published review of studies using data-driven approaches to investigate associations between prescribed medications and dementia risk pointed out reverse causality as a plausible explanation for the increased dementia risk observed in users of antidepressants and antipsychotics [41]. Moreover, PIMcogs encompass a wide range of drug classes, and further studies using data-driven approaches to identify individual candidates that influence cognitive decline and dementia risk are needed. The link between long-term exposure to benzodiazepines, -particularly diazepam- and dementia, has been the most extensively investigated; however, a causal connection through potential pathogenic pathways has yet to be demonstrated [16]. Additional studies are required to understand the underlying mechanisms of the long-term effects of benzodiazepines and anticholinergic drugs on cognitive decline, as well as to establish any plausible biological pathways linking cumulative PIMcog exposure to neurodegenerative processes in individuals with cognitive disorders.

We chose to identify PIMcogs using Beers Criteria 2019 and STOPP version 2. A general limitation of defining drugs as inappropriate based solely on generic drug lists is the lack of information about individual’s clinical drug responses, especially in populations with significant interindividual variability. Drugs identified as PIMcogs may be necessary, well-tolerated, and clinically appropriate for some individuals, while the same drug and dosage may be considered inappropriate for others. Accordingly, the interpretation of our results is limited by the absence of data on patients’ comorbidities, intercurrent diseases, and actual drug responses. Additionally, the retrospective study design precluded obtaining clinical information about the reasons for prescribing PIMcogs, and we were unable to identify whether specific patient profiles are more likely to receive PIMcogs or how this relates to their overall symptomatology. Another limitation to the generalizability of our results is the selection bias caused by the inclusion of 62 participants from the YOD study causing a low median age in our study cohort. The recording of drugs is based on self-reported use given in an interview setting at the memory clinics. Previous research has shown that there is a considerable unreported use of psychotropic drugs on admission to hospital [42]. Many of the PIMcogs are used *pro re nata*, and for these reasons, we have likely underestimated the use of PIMcogs in this study. Finally, we were not able to record the dosages of the prescribed PIMcogs which are of importance in terms possible risks of drug-induced acceleration of cognitive decline and disease progression.

The present study provides new insights into the association between the use of potentially inappropriate medications and cognitive deterioration and disease progression in home-dwelling individuals with MCI and early-stage dementia. Strengths of this study include the enrollment of patients from eight memory clinics across multiple counties in Norway, representing a broad range of cognitive diagnoses and disease severities. Additionally, the novelty of this study is strengthened by the inclusion of several measures for disease progression and its large cohort of individuals with young-onset cognitive disorders, a group that has not been extensively studied in relation to inappropriate drug use and disease progression.

## Conclusion

The prevalence of PIMcogs and the overall drug use were lower in patients attending Norwegian memory clinics than reported from memory clinics in other countries. Still, almost one fourth used medications that were associated with faster dementia progression. The impact of PIMcogs on specific patient profiles and how this relates to their overall symptomatology needs further research. However, the provided knowledge could help inform safer treatment approaches for dementia patients and invites to a more critical look at prescribing patterns and their implications for patient care.

## Supplementary Information

Below is the link to the electronic supplementary material.

ESM 1 (PDF 167 KB)

## Data Availability

No datasets were generated or analysed during the current study.

## Declarations

### Prior presentation

The study was presented in an oral communication session at the 20th EuGMS Congress, Valencia, 2024. All tables, figures and exact numbers were left out of the oral presentation.

### Registration in

https://www.clinicaltrials.gov/:Clinical trial number: not applicable*.*
