# Supplementary material for: Potentially inappropriate medications related to two-year progression of mild cognitive impairment and dementia
Source: Eur J Clin Pharmacol. 2026 Jan 19;82(2):45. doi: 10.1007/s00228-025-03977-6 (PMC12816031; doi:10.1007/s00228-025-03977-6)
Supplement: Supplementary file 1 — (PDF 167 KB) [file 228_2025_3977_MOESM1_ESM.pdf]

Appendix 1: List of PIMcogs and frequency of use (#) in 397 outpatients attending Norwegian memory clinics

Drug substance	ATC code	#	#	Drug substance	ATC code	#	#	Drug substance	ATC code	#	#
**Anticholinergics**		**baseline**	**follow-up**	**Hypnotics/ benzodiazepines**		**baseline**	**follow-up**	**Antipsychotics**		**baseline**	**follow-up**
Atropine	A03BA01	0	0	Midazolam	N05CD08	0	0	Haloperidol	N05AD01	0	2
Butylscopolamine	A04AD01	0	0	Alprazolam	N05BA12	0	0	Sertindole	N05AE03	0	0
Disopyramide	C01BA03	0	0	Klometiazol	N05CM02	0	4	Ziprasidone	N05AE04	0	0
Oxybutynin	G04BD04	0	1	Deksmedetomidin	N05CM18	0	0	Lurasidone	N05AE05	0	0
Tolterodine	G04BD07	4	2	Zopiclone	N05CF01	21	35	Flupentixol	N05AF01	0	1
Solifenacin	G04BD08	9	9	Oxazepam	N05BA04	9	17	Zuclopenthixol	N05AF05	0	0
Darifenacin	G04BD10	3	1	Zolpidem	N05CF02	1	5	Quetiapine	N05AH04	3	8
Fesoterodine	G04BD11	3	0	Diazepam	N05BA01	0	5	Amisulpride	N05AL05	0	0
Biperiden	N04AA02	0	0	Nitrazepam	N05CD02	0	1	Lithium	N05AN01	2	1
Perphenazine	N05AB03	0	0	Lorazepam	N05BA06	0	0	Risperidone	N05AX08	3	11
Prochlorperazine	N05AB04	1	0					Aripiprazole	N05AX12	1	1
Chlorprothixene	N05AF03	0	2					Paliperidone	N05AX13	0	0
Clozapine	N05AH02	0	0					Cariprazine	N05AX15	0	0
Olanzapine	N05AH03	2	6					Brexpiprazole	N05AX16	0	0
Hydroxyzine	N05BB01	1	3								
Levomepromazine	N05AA02	3	1								
Paroxetine	N06AB05	0	0								
Clomipramine	N06AA04	0	0								
Trimipramine	N06AA06	1	1								

Amitriptyline	N06AA09	6	4
Nortriptyline	N06AA10	0	0
Mebhydroline	N06AA12	1	0
Dexchlorpheniramine	R06AB02	0	0
Alimemazine	R06AD01	1	4
Promethazine	R06AD02	0	1
Cyclizine	R06AE03	0	0
Meclozine	R06AE05	0	1
Diphenhydramine	R06AA02	0	0
Doxylamine	R06AA09	0	0
